# Effect of false positive and false negative rates on inference of binding target conservation across different conditions and species from ChIP-chip data

**DOI:** 10.1186/1471-2105-10-23

**Published:** 2009-01-19

**Authors:** Debayan Datta, Hongyu Zhao

**Affiliations:** 1 Department of Biomedical Engineering, Yale University, New Haven, CT 06520, USA; 2 Department of Epidemiology and Public Health, Yale University, New Haven, CT 06520, USA; 3 Department of Genetics, Yale University, New Haven, CT 06520, USA

## Abstract

**Background:**

ChIP-chip data are routinely used to identify transcription factor binding targets. However, the presence of false positives and false negatives in ChIP-chip data complicates and hinders analyses, especially when the binding targets for a specific transcription factor are compared across conditions or species.

**Results:**

We propose an Expectation Maximization based approach to infer the underlying true counts of "positives" and "negatives" from the observed counts. Based on this approach, we study the effect of false positives and false negatives on inferences related to transcription regulation.

**Conclusion:**

Our results indicate that if there is a significant degree of association among the binding targets across conditions/species (log odds ratio > 4), moderate values of false positive and false negative rates (0.005 and 0.4 respectively) would not change our inference qualitatively (i.e. the presence or absence of conservation) based on the observed experimental data despite a significant change in the observed counts. However, if the underlying association is marginal, with odds ratios close to 1, moderate to large values of false positive and false negative rates (0.01 and 0.2 respectively) could mask the underlying association.

## Background

Transcription factors play an important role in gene regulation by binding to specific DNA sequences in the regulatory regions of their targets. Accurate identification of the binding targets of the transcription factors is paramount to the understanding of the regulatory mechanism. Chromatin immunoprecipitation (ChIP) experiments are commonly used to identify the regulatory targets in prokaryotes and eukaryotes. ChIP-chip experiments provide us with information about the binding targets of a particular regulator at the genome level [1-4].

The output of ChIP-chip experiments are often summarized in binary forms. Using replicate data, the statistical evidence for a gene being the binding target of a transcription factor is typically summarized as a p-value. A threshold for the p-value, e.g. 0.001 is then chosen, and genes with p-values less than the threshold are considered the binding targets for the transcription factor. Thus, for a transcription factor, we can enumerate a list of genes which are "positives", i.e. binding targets and a list of genes which are "negatives", i.e. non-binding targets. If the threshold is set at a very stringent level to control the number of false positives, this will be achieved at the expense of high false negatives. A more relaxed threshold will reduce the number of false negatives, but will end up with more false positive results. Over the past few years, ChIP-chip data has formed the basis of many transcription regulatory mechanism studies. Several groups have compared the binding of a regulator across multiple experimental conditions to determine condition dependence of binding [5-7]. Similarly, binding data of specific transcription factors across species has been used to investigate the presence of conserved binding targets [8]. Unfortunately, the presence of noise, in the form of false positives and false negatives as discussed above, may lead to inaccurate inference of the binding targets, and thus biased results and potentially incorrect conclusions on key aspects of transcription regulation, e.g. preservation of regulation targets across conditions and species. In this article, we develop a statistical approach to analyzing ChIP-chip data, appropriately incorporating false positives and false negatives. Based on our approach, we investigate the effect of false positives and false negatives on the inference of conservations of binding targets based on ChIP-chip data.

## Methods

### Summarizing Contingency Tables

As discussed above, the output of ChIP-chip experiments is typically summarized into binary forms and results across different experiments for the same transcription factor can be crosstabulated into a contingency table. A common question asked is whether a transcription factor has similar binding targets across conditions, and this is reflected as the dependency of outcome among the conditions. In the following, we give a brief discussion on two statistical measures that we will use to summarize the degree of dependency in a contingency table.

For the sake of clarity, we will focus on ChIP-chip experiments involving two different conditions or two species. The number of target genes in the two conditions/species can be cross tabulated into a 2 by 2 contingency table. We use two metrics to summarize such contingency tables – *Odds Ratio *and *Positive specific agreement *[9,10].

Table 1 gives an illustration of a 2 by 2 contingency table. The goal is to identify whether a relationship, or association exists between the two categorical variables. In our scenario, it would correspond to whether the transcription factor exhibits condition dependent binding or condition independent binding. For such a contingency table, the *odds ratio *is a commonly used measure to quantify association among the categorical variables. An odds is defined as the ratio of the frequency of being in one category and the frequency of not being in that category. For example, from Table 1, the odds that a particular gene is a binding target in experimental condition 1 is equal to (*c *+ *d*)/(*a *+ *b*). This odds is called marginal odds, obtained from the total frequencies in one margin of the table, disregarding the effects of the other variable. Conditional odds are the chances of the transcription factor binding relative to not binding in one experimental condition, given a particular level (binding state) in the other experimental condition. The variables are deemed to be unassociated if the conditional odds are equal or close to each other, and hence equal to the marginal odds. To compare directly the two conditional odds, a single summary statistic, obtained by dividing the first conditional odds by the second is called odds ratio. Thus, for the data in Table 1, the odds ratio is defined as: Odds Ratio = (*a*/*c*)/(*b*/*d*) = *ad*/*bc*. Odds ratio takes only positive values and has no upper limit. An odds ratio of 1 indicates no relationship among the variables. In addition to the odds ratio, its logarithm is also commonly used. Logarithmic transformation of data has a number of advantages – the variation of log transformed data tends to be less dependent on the magnitude of values, while taking logs also reduces the skewness of the distributions. After log transformation, data tends to be spread out more evenly, also making it easier to examine visually.

**Table 1:** Simple 2 by 2 contingency table.

	**Condition 2**		
	
		0	1
	
**Condition 1**	0	a	b
	
	1	c	d

The categorical variables Condition 1 and Condition 2 each has two levels. The cell values are counts at each combination of two condition variables.

Other measures of dependency are also often used in psychological and medical research. For example, the problem can be formulated as follows: Suppose two raters classify each subject in a sample from some target population according to the presence or absence of some characteristic of interest. The resulting data can then be summarized into a 2 by 2 table. The agreement between raters can be quantified by the metric *simple agreement*, which is defined as the proportion of cases for which both raters agree, or (*a *+ *d*)/(*a *+ *b *+ *c *+ *d*). However, if *a *is large, this would approach 1 regardless of the performance on positive cases. *Positive specific agreement *provides insight when the positive cases are rare. It estimates the conditional probability that one rater will agree that a case is positive given the other one rated it positive, where the role of the two raters is selected randomly. Positive specific agreement, *p*_*pos *_is defined as: *p*_*pos *_= 2*d*/(2*d *+ *b *+ *c*). Both (log) odds ratio and positive specific agreement will be considered in our following discussion.

### Model Setup

Consider an experiment with a binary outcome. Let *p*_0 _denote the proportion of true negatives, while *p*_1 _be the proportion of true positives. We denote **p **= (*p*_0_, *p*_1_)^*t *^as the vector of true proportions. Due to false positives and false negatives, the observed proportions likely differ from the true proportions. Let $\hat{p}={\left({\hat{p}}_{0},{\hat{p}}_{1}\right)}^{t}$ denote the vector of the observed proportions. The relationship between **p **and *E*($\hat{p}$) can be written as:

(1) $$\left(\begin{array}{c} E({\hat{p}}_{0})\\ E({\hat{p}}_{1}) \end{array}\right)=\left(\begin{array}{cc} 1-s & t\\ s & 1-t \end{array}\right)\left(\begin{array}{c} p_{0}\\ p_{1} \end{array}\right),$$

where *s *is the false positive rate and *t *is the false negative rate. Denoting the transformation matrix as *M*, Equation (1) can be written as:

(2) $$E(\hat{p})=Mp.$$

Thus, for different values of false positive and false negative rates, different observed proportions will be obtained based on Equation (2). If the false positive and false negative rates are known, the true proportions may be inferred based on the observed experimental proportions. Multiplying both sides of Equation (2) by *M*^-1 ^gives us:

(3) $$M^{-1}E(\hat{p})=p.$$

However, due to chance variations, **p **obtained through this approach based on the observed $\hat{p}$ may have negative components, leading to uninterpretable results. Instead, we propose to estimate the true proportions using an Expectation Maximization (EM) based approach explained in detail in the following subsection.

Often, we are interested in the analysis of the binding of a particular transcription factor in multiple experimental conditions or across different species. In either case, we are interested in counts of similarity of binding across conditions or organisms. This would correspond to an extension of Equation (2) into a higher dimension. For simplicity, we present our analysis for a 2-dimensional case. For example, if we consider the binding targets of a transcription factor across two experimental conditions, the vector of true proportions can be represented as **p **= (*p*_00_, *p*_01_, *p*_10_, *p*_11_)^*t*^. Here *p*_00 _denotes the proportion of genes which are not targets of the regulator in either condition, *p*_01 _denotes the proportion of genes which are targets of the regulator in the second condition but not in the first, *p*_10 _denotes the proportion of genes which are targets of the regulator in the first condition but not in the second, while *p*_11 _denotes the proportion of genes which are targets of the regulator in both conditions. Similarly, the vector for the observed proportions can be denoted as $\hat{p}={\left({\hat{p}}_{00},{\hat{p}}_{01},{\hat{p}}_{10},{\hat{p}}_{11}\right)}^{t}$. The relationship between the observed and true proportions can be then written as:

(4) $$E(\hat{p})=(M⊗M)p.$$

If we consider Equation (2) to correspond to a 1-dimensional case, for the *n*-dimensional case, the new transformation matrix would simply be obtained by taking the tensor product of *M *with itself *n *times. Here we assume that the false positive and false negative rates to be the same across two conditions. In general that may not be the case. In such a scenario, for a 2-dimensional case, Equation 4 takes the general form:

(5) $$E(\hat{p})=(M_{1}⊗M_{2})p,$$

where *M*_1 _and *M*_2 _are the transformation matrices for the first and second conditions respectively.

### EM Algorithm

Given a vector of observed proportions which we obtain from experimental output, for different values of false positive rates and false negative rates, we aim to infer the true proportions. This would give us an idea about how the observed and true proportions differ for different levels of noise in the form of false positives and false negatives. We infer the true proportions from the observed proportions using an EM based approach which we now discuss in detail.

Let us consider the binding patterns for a transcription factor in experimental conditions *c*_1 _and *c*_2_. We define the vector for the true binary binding pattern of a particular gene *G *as **b **= (*b*_1_, *b*_2_), where *b*_1 _and *b*_2 _take binary value 1 or 0 depending on whether the gene is a true binding target for the transcription factor in *c*_1 _and *c*_2 _respectively. Thus, for the experimental conditions *c*_1 _and *c*_2_, this binary binding pattern vector can take *four *possible values, {(0, 0), (0, 1), (1, 0), (1, 1)}. For example, a binary binding pattern vector equal to (1, 1) indicates that the gene is a binding target for the transcription factor in both *c*_1 _and *c*_2_. We aim to infer this true binary binding pattern for all the genes and thus obtain the true binary counts. Due to experimental errors, we have the observed counts as the experimental output.

We denote the observed binding pattern for a particular gene as **g **= (*g*_1_, *g*_2_), where each component is either 0 or 1 denoting whether the gene is observed to be the binding target of the particular transcription factor in *c*_1 _and *c*_2 _respectively, based on the experimental output. Thus, the vector **g **represents the observed data. The probability of the observed binding data is then given by

(6) $$\begin{array}{lll} P(g) & = & P(b=(0,0))P(g|b=(0,0))+P(b=(0,1))P(g|b=(0,1))+\\ & & P(b=(1,0))P(g|b=(1,0))+P(b=(1,1))P(g|b=(1,1)). \end{array}$$

Thus, for *N *genes, the probability of the observed data is

(7) $$P(g_{1},g_{2},...,g_{N})=\prod_{i=1}^{N}P(g_{i}).$$

In this article, we propose to estimate the *P*(**b**) to maximize *P*(**g**_1_, **g**_2_, ..., **g**_*N*_) using the EM algorithm, by treating **b **as the missing data as follows.

**E-Step**: In the Expectation step, the conditional distribution of the missing data given the observed data is evaluated. We evaluate the posterior probabilities of each true binding state given the observed binding pattern. Thus, for every gene *G *with observed binding pattern **g**, we estimate:

(8) $$P(b^{\left(m\right)}|g)=\frac{P(g|b^{\left(m\right)})*P(b^{\left(m\right)})}{\sum P(g|b^{\left(m\right)})*P(b^{\left(m\right)})}$$

where **b**^(*m*) ^is the estimate of the true binding state **b **probability at the m-th step. Since **b**^(*m*) ^can have four possible values, at each step, we estimate four probabilities. The probability *P*(**g**|**b**^(*m*)^) can be expanded as:

(9) $$\begin{array}{lll} P(g|b^{\left(m\right)}) & = & P((g_{1},g_{2})|(b_{1}^{\left(m\right)},b_{2}^{\left(m\right)}))\\ & = & P(g_{1}|b_{1}^{\left(m\right)})P(g_{2}|b_{2}^{\left(m\right)}), \end{array}$$

where $b_{1}^{\left(m\right)}$ and $b_{2}^{\left(m\right)}$ are the first and second components of the estimate **b**^(*m*) ^and take binary value 0 or 1.

The second equation in (9) results from the independence assumption for the data from two separate ChIP-chip experiments. Thus, the probability of observing *g*_1 _would be independent of the estimate of binding state $b_{2}^{\left(m\right)}$, while the probability of observing *g*_2 _would be independent of the estimate of binding state $b_{1}^{\left(m\right)}$. There are four possible cases for the expression $P(g_{i}|b_{i}^{\left(m\right)})$ in Equation (4). From Equation (1), they can be enumerated as:

(10) $$\begin{array}{ll} P(g_{i}=0|b_{i}^{\left(m\right)}=0)=1-s, & P(g_{i}=0|b_{i}^{\left(m\right)}=1)=t,\\ P(g_{i}=1|b_{i}^{\left(m\right)}=0)=s, & P(g_{i}=1|b_{i}^{\left(m\right)}=1)=1-t. \end{array}$$

Thus, for each gene, we start with a set of estimates *P*(**b**^(*m*)^) and obtain estimates of the posterior probabilities *P*(**b**^(*m*)^|**g**) for each gene at the E-step.

**M-Step**: In the Maximization step, the parameters *P*(**b**) are re-estimated to maximize the likelihood of the complete data. After obtaining *P*(**b**^(*m*)^|**g**) for each gene, we cross-tabulate a two-way contingency table, with the "count" for each of the four values {(0, 0), (0, 1), (1, 0), (1, 1)} being the sum of the probabilities for that particular value across all the genes. These counts are then used to obtain updated P estimates for *P*(**b**). For example, $P(b=(0,0))=\frac{1}{N}\sum P(b=0,0)|g_{i})$.

We iterate between the E-Step and the M-Step until convergence. The convergence criterion was set as: |*P*(**b**^(*m*)^) - *P*(**b**^(*m*-1)^)| < 10^-12^.

## Results and discussion

In this section, we study the effect of false positives and false negatives on inferring regulatory target conservation across conditions/species through both simulations and real data analyses.

**Figure 1:**
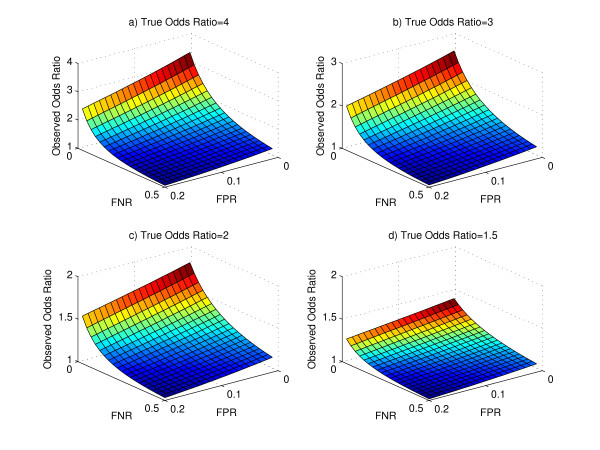
**Surface of the Observed Odds Ratio for different values of false positive rate and false negative rate and different values of True Odds Ratio**. We observe that the surface is highest for low values of the false positive rate and false negative rate, and falls for increasing values of the false positive rate and false negative rate.

**Figure 2:**
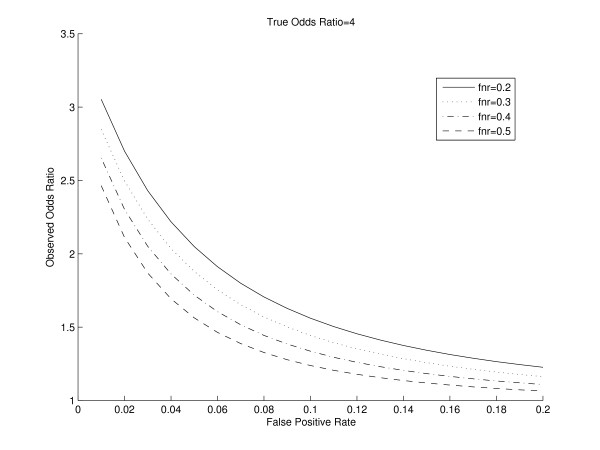
**Plot of the Observed Odds Ratio versus the false positive rate for different values of false negative rate and fixed True Odds Ratio**. The Observed Odds Ratio decreases with increasing false positive rate for a fixed false negative rate.

We consider an experiment involving the binding of a transcription factor in two different conditions with a total of 1000 genes. We consider the odds ratio as our metric of interest. For fixed true odds ratios, and different values of false positive and false negative rates, we plot the surface of the observed odds ratio in Figure 1. The observed odds ratio is obtained from Equation 4. It can be seen that the observed odds ratio is the largest for low values of false positive and false negative rates and its value decreases with increasing false positive and false negative rates. To visualize this phenomenon in two-dimensions, we fix the false negative rate, and plot the observed odds ratio as the false positive rate varies (Figure 2). We observe that with increasing false positive rates, the observed odds ratio decreases. This is expected, as with an increasing false positive rate, a larger number of true negatives are detected as positives. This reduces the count of genes which are observed negatives in both conditions, i.e. the cell in the contingency table corresponding to "00". Thus, there is a reduction in the observed odds ratio value. Similarly, we observe that for a fixed false positive rate with an increasing false negative rate, the observed odds ratio also decreases. This is also expected, as with increasing false negative rate, a larger number of true positives are detected as negatives. This reduces the number of genes which are observed positives in both conditions, i.e. the cell in the contingency table corresponding to "11", thereby causing a reduction in the observed odds ratio value. To study the effect of asymmetry between *p*_01 _and *p*_10_, we repeated this simulation for differing values of *p*_01 _and *p*_10_. We observed similar trends of decreasing observed odds ratios for increasing false positive rate for a fixed false negative rate and a fixed true odds ratio.

In the following, we give an analytical proof for the reduction in the observed odds ratio for increasing false positive rates, with the false negative rate being fixed. Equation 4 can be expanded as:

(11) $$\left(\begin{array}{c} E({\hat{p}}_{00})\\ E({\hat{p}}_{01})\\ E({\hat{p}}_{10})\\ E({\hat{p}}_{11}) \end{array}\right)=\left(\begin{array}{cccc} {\left(1-s\right)}^{2} & (1-s)t & t(1-s) & t^{2}\\ (1-s)s & \left(1-s)(1-t\right) & ts & t(1-t)\\ s(1-s) & st & \left(1-t)(1-s\right) & (1-t)t\\ s^{2} & s(1-t) & (1-t)s & {\left(1-t\right)}^{2} \end{array}\right)\left(\begin{array}{c} p_{00}\\ p_{01}\\ p_{10}\\ p_{11} \end{array}\right).$$

The observed odds ratio is:

(12) $$OOR=\frac{E({\hat{p}}_{00})*E({\hat{p}}_{11})}{E({\hat{p}}_{01})*E({\hat{p}}_{10})}$$

where from Equation 11 we get,

$$\begin{array}{l} E({\hat{p}}_{00})=p_{00}{\left(1-s\right)}^{2}+(p_{01}+p_{10})(1-s)t+p_{11}t^{2},\\ E({\hat{p}}_{01})=p_{00}(1-s)s+p_{01}(1-s)(1-t)+p_{10}st+p_{11}(1-t)t,\\ E({\hat{p}}_{10})=p_{00}(1-s)s+p_{01}st+p_{10}(1-s)(1-t)+p_{11}(1-t)t,\\ E({\hat{p}}_{11})=p_{00}s^{2}+(p_{01}+p_{10})s(1-t)+p_{11}{\left(1-t\right)}^{2}. \end{array}$$

We show that for a true odds ratio greater than 1 and for *s *< 1/2 and *s *+ *t *< 1, ∂ (*OOR*)/∂*s *is negative. These are reasonable assumptions for real data, where the false positives are low and false negatives are not very high. The denominator of ∂ (*OOR*)/∂*s *is always positive as it is a squared number. The numerator can be written as:

*F *= *F*1 * F2 * F3

where,

*F*1 = (*p*_01_*p*_10 _- *p*_00_*p*_11_)(-1 + *s *+ *t*),

*F*2 = (1 - *s*)(*p*_01 _+ *p*_10 _+ 2*p*_00_*s*) + (-*p*_01 _- *p*_10 _+ 2*p*_11 _+ 2(*p*_01 _+ *p*_10_)*s*)*t *- 2*p*_11_*t*^2^,

*F*3 = (1 - *p*_00_)(-1 + *t*)*t *+ *p*_00_(-1 + *t *- *s*(-2 + *s *+ 2*t*)).

Let us consider each term separately.

If the true odds ratio is greater than 1 and *s *+ *t *< 1, then *p*_01_*p*_10 _<*p*_00_*p*_11 _and (-1 + *s *+ *t*) < 0. Thus, we have *F*1 > 0. *F*2 can be simplified as:

*F*2 = (1 - *s*)(*p*_01 _+ *p*_10 _+ 2*p*_00_*s*) + (*p*_01 _+ *p*_10_)(-1 + 2*s*)*t *+ 2*p*_11_*t*(1 - *t*).

Thus, if (-1 + 2*s*) < 0, i.e. *s *< 1/2, then all the three product terms in *F*2 are positive. Thus, for *s *< 1/2, *F*2 > 0. *F*3 can be simplified as:

*F*3 = (-1 + *t*)(*t*(1 - *p*_00_) + *p*_00_) - *p*_00_*s*(-2 + *s *+ 2*t*) = -*p*_00_*s*^2 ^+ 2*p*_00_*s*(1 - *t*) + (-1 + *t*)(*t*(1 - *p*_00_) + *p*_00_).

Thus, *F*3 is a quadratic function of *s*. Since the coefficient of *s*^2 ^is negative, if the discriminant of the quadratic is negative, *F*3 is always negative. The discriminant *D *is given by:

$$D=4p_{00}^{2}{\left(1-t\right)}^{2}+4p_{00}(-1+t)(t(1-p_{00})+p_{00})$$

Simplifying, we get, $D=-4p_{00}^{2}(1-t)t-4p_{00}(1-t)t(1-p_{00})$ which is clearly negative. Thus, *F *is negative for true odds ratio greater than 1, *s *< 1/2 and *s *+ *t *< 1.

### Simulation Results

**Figure 3:**
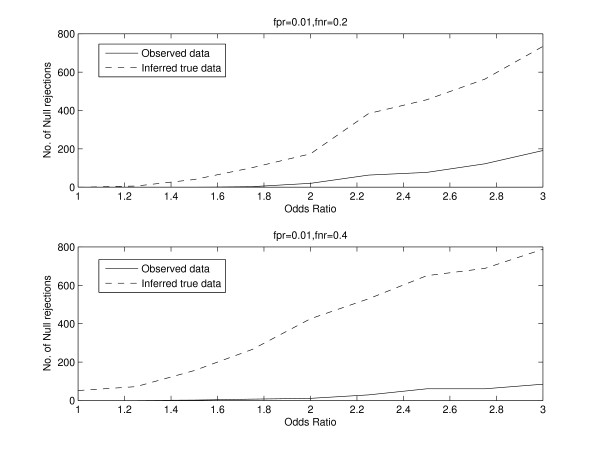
**Simulation results showing the plot of the number of null rejections versus the odds ratio for both the observed and inferred true data**. The number of null rejections for the inferred true data is consistently larger than that for the observed data.

**Figure 4:**
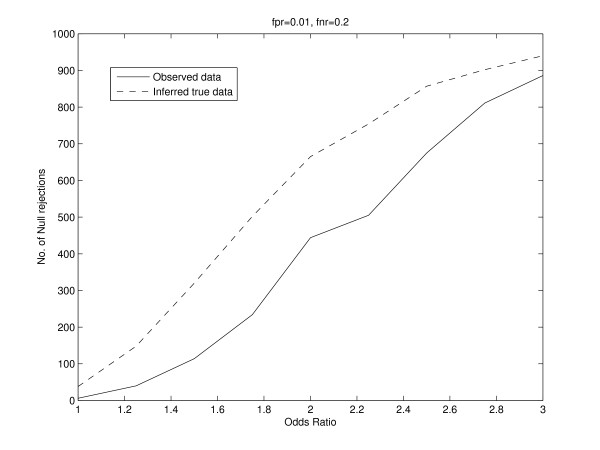
**Simulation results showing the plot of the number of null rejections versus the odds ratio for both the observed and inferred true data**. The number of null rejections are obtained by applying a threshold of 1.5 to the *Odds ratio*. The number of null rejections for the inferred true data is consistently larger than that for the observed data.

**Figure 5:**
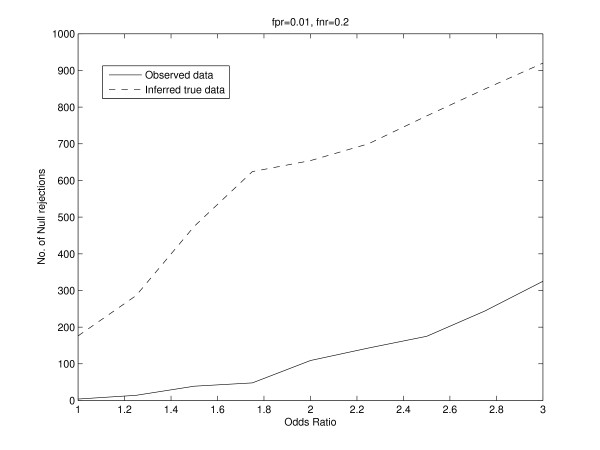
**Simulation results showing the plot of the number of null rejections versus the odds ratio for both the observed and inferred true data**. The number of null rejections are obtained by applying a threshold of 0.15 to the *Positive specific agreement*. The number of null rejections for the inferred true data is consistently larger than that for the observed data.

To study the effect of false positives and false negatives on statistical inference of dependence between two conditions, we consider a similar setting in which the binding of a regulator in two conditions is studied for 1000 genes. We simulated data for a fixed true odds ratio, and fixed the false positive and false negative rates. We randomly added false positives and false negatives to the data based on the false positive rate and false negative rate. This manifests itself as the observed data, and we repeated this 1000 times. We performed a chi-squared test for independence between the two conditions and counted the number of times the null hypothesis was rejected at a significance level of 0.001. Further, for each observed dataset, we inferred back the true data using our EM algorithm. The inferred true counts are almost equal to the true counts before false positives and false negatives were randomly added. This is because the false positives and false negatives were randomly added based on the fixed false positive rate and false negative rate. These fixed rates are used in our EM algorithm to obtain the inferred true counts. For example, for a true odds ratio of 2, the vector of true counts was (800, 100, 80, 20)^*t*^. We fixed the false positive rate to 0.01 and the false negative rate to 0.2. The vector of inferred true counts was determined to be (799.87, 101.92, 78.55, 20.66)^*t*^. The EM algorithm was initialized by giving equal weights to each possible true binding pattern for each gene. We used the chi-squared test and counted the number of times the null hypothesis was rejected for the inferred true data at the same level of significance. We repeated this analysis for different values of the true odds ratio and different values of false positive and false negative rates. Figure 3 shows the plot of the number of null rejections versus the odds ratio for both the observed and inferred true data. Our results indicate that the number of null rejections for the inferred true data is consistently larger than that for the observed data. We also note that as the odds ratio increases, the difference between the number of null rejections for the inferred true data and observed data also increases. Instead of using the chi-squared test, we also used used thresholds for the odds ratio and positive specific agreement to ascertain the number of null rejections for observed and inferred true data. Thus, for each simulation, we rejected the null hypothesis if the odds ratio was greater than some threshold. We repeated this by applying a threshold to the positive specific agreement. The results are shown in Figures 4 and 5. Thus, in addition to the chi-squared, thresholds for the odds ratio and positive specific agreement also provide evidence that the number of null rejections for the inferred true data is consistently larger than that for the observed data.

### Real Datasets

We considered two ChIP-chip datasets. Harbison *et al. *[5] described the binding profiles of 204 transcription factors for *S. Cerevisiae *in Rich medium, and 84 of these transcription factors were also profiled in at least one other experimental condition. In their study, transcription factors were selected for profiling in a particular environment if they were essential for growth in that environment, or if there was other evidence suggesting their role in gene regulation in that environment. Borneman *et al. *[8]. studied the divergence of binding sites of regulators Ste12 and Tec1 in the yeasts *S. cerevisiae*, *S. mikatae *and *S. bayanus *under pseudohyphal conditions. They listed genes which showed differing degrees of conservation across the three species, i.e. genes which were targets in only one species, the targets in two species, and the targets all three species.

For ChIP-chip data from Harbison *et al. *[5], we focussed on the binding data for the transcription factors Ste12 and Tec1 in three different experimental conditions – Rich medium, Filamentation inducing and Mating inducing. We used a p-value threshold of 0.001 to obtain the binding targets for these two regulators. For a pair of experimental conditions, we cross-tabulated the binding targets and created a 2 by 2 contingency table. The odds ratio was quite high, hence we used the log odds ratio and positive specific agreement as metrics to summarize the contingency tables. Thus, given the observed log odds ratio and observed positive specific agreement, for different values of the false positive rate and false negative rate, we inferred the underlying true log odds ratio and the true positive specific agreement using our EM based approach.

In the section describing the model setup, we stated that multiplication of the vector of the observed proportions with the inverse of the transformation matrix could lead to inferred true proportions with negative components. Here we illustrate the scenario. For the regulator Ste12, in Rich Medium and Mating inducing condition the vector of the observed proportions is **p **= (0.9761, 0.0144, 0.0040, 0.0056)^*t*^. For a false positive rate of 0.001 and a false negative rate of 0.2, multiplying **p **by the inverse of the transformation matrix results in the vector of the inferred true proportions $\hat{p}$ = (0.9743, 0.0150, 0.0020, 0.0087)^*t*^. However, for a false positive rate of 0.002 and a false negative rate of 0.3, the vector of the inferred true proportions is $\hat{p}$ = (0.9748, 0.0144, -0.0005, 0.0113)^*t*^. Similarly, for a false positive rate of 0.004 and a false negative rate of 0.4, the vector of the inferred true proportions is $\hat{p}$ = (0.9793, 0.0113, -0.0061, 0.0154)^*t*^. Thus, the inferred true proportions obtained by simply multiplying the observed proportions with the inverse of the transformation matrix could contain negative components.

**Figure 6:**
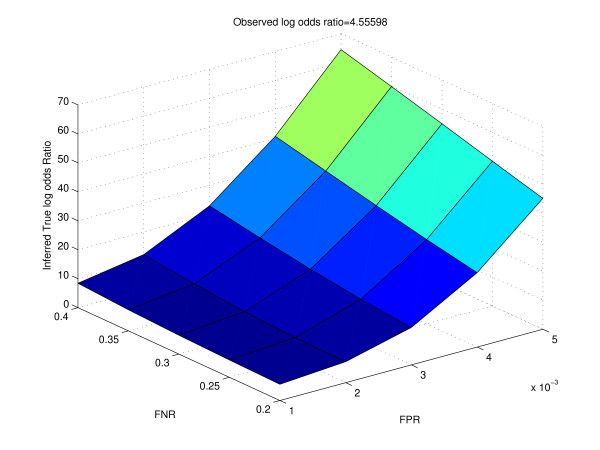
**Surface of the inferred true log odds ratio for different values of false positive rate and false negative rate for real data**. Observed log odds ratio is obtained from cross-tabulation of the binding targets of Ste12 in Rich Medium and Mating Inducing condition.

**Figure 7:**
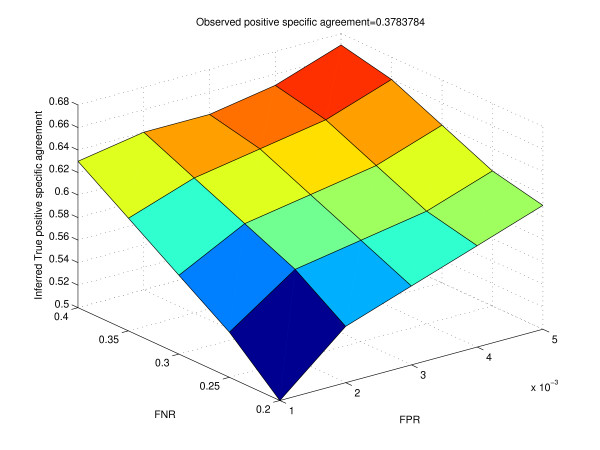
**Surface of the inferred positive specific agreement for different values of false positive rate and false negative rate for real data**. Observed positive specific agreement value is obtained from cross-tabulation of the binding targets of Ste12 in Rich Medium and Mating Inducing condition.

Table 2 shows how the inferred true proportions change for different values of the false positive rate and false negative rate. The vector of the observed proportions is (0.976, 0.014, 0.004, 0.006)^*t*^. Table 3 gives the calculation of the inferred true odds ratios from the inferred true proportions. Figure 6 shows how the surface of the inferred true log odds ratio varies with different values of false positive rate and false negative rate. We notice that as the false positive rate and false negative rate increase, the inferred true log odds ratio differs quite significantly from the observed log odds ratio. We observe similar trends when we use positive specific agreement as the metric of interest (Table 4 and Figure 7). Harbison *et al. *reported that the false discovery rate in their data was likely to be approximately 4%, while the false negative rate was around 24% for a p-value threshold of 0.001. For binding data from Harbison *et al.*, typically the number of "negatives" was close to 6000, while the number of "positives" was about 100 to 200 at a p-value threshold of 0.001. Thus, the false positive rate was close to 0.001. For our analysis, we studied the variation of observed and true outcomes by varying the false positive rate from 0.001 to 0.005, and the false negative rate from 0.2 to 0.4. Thus, our range of false positive rates would correspond to about 6 to 30 false positives, and about 20 (100 * 0.2) to 80 (200 * 0.4) false negatives, which appears to be quite reasonable. From Table 3, we see that for a false positive rate of 0.001 and false negative rate of 0.20, the inferred true log odds ratio is 5.60, while the observed log odds ratio is 4.56. Since both the log odds ratios are quite high, our inference of association among the two experimental conditions would not be affected by these values of the false positive rate and false negative rate.

**Table 2:** Inferred true proportions of target genes of Ste12 in the Rich Medium and Mating Inducing conditions.

	**FNR**				
	**0.20**	**0.25**	**0.30**	**0.35**	**0.40**
	
**FPR = 0.001**	0.974	0.973	0.972	0.971	0.969
	0.015	0.015	0.016	0.106	0.017
	0.002	0.002	0.001	0.001	0.001
	0.009	0.010	0.011	0.013	0.014

	**0.20**	**0.25**	**0.30**	**0.35**	**0.40**
	
**FPR = 0.002**	0.977	0.976	0.974	0.973	0.971
	0.014	0.014	0.014	0.015	0.015
	0.001	0.001	0.001	0	0
	0.009	0.010	0.011	0.012	0.013

	**0.20**	**0.25**	**0.30**	**0.35**	**0.40**
	
**FPR = 0.003**	0.979	0.978	0.976	0.975	0.973
	0.013	0.013	0.013	0.014	0.014
	0	0	0	0	0
	0.009	0.009	0.010	0.011	0.013

	**0.20**	**0.25**	**0.30**	**0.35**	**0.40**
	
**FPR = 0.004**	0.980	0.979	0.978	0.977	0.975
	0.011	0.012	0.012	0.012	0.013
	0	0	0	0	0
	0.008	0.009	0.010	0.011	0.012

	**0.20**	**0.25**	**0.30**	**0.35**	**0.40**
	
**FPR = 0.005**	0.982	0.981	0.980	0.979	0.977
	0.010	0.011	0.011	0.011	0.011
	0	0	0	0	0
	0.008	0.009	0.010	0.010	0.012

For different values of false positive and false negative rates, the four inferred proportions of the target genes of Ste12 in Rich Medium and Mating Inducing are tabulated. The observed vector of proportions is (0.976, 0.014, 0.004, 0.006)^*t*^.

**Table 3:** Inferred true log odds ratios for target genes of Ste12 in the Rich Medium and Mating Inducing conditions.

	**FNR**					
	
		**0.20**	**0.25**	**0.30**	**0.35**	**0.40**
	
**FPR**	**0.001**	5.60	5.98	6.50	7.28	8.44
	
	**0.002**	7.24	7.55	8.54	10.11	12.18
	
	**0.003**	12.93	14.63	16.98	19.78	22.88
	
	**0.004**	25.68	29.10	32.83	36.74	40.73
	
	**0.005**	45.21	50.03	54.92	59.77	64.51

Inferred true log odds ratio values for different values of false positive rate and false negative rate for the binding targets of Ste12 in the Rich Medium and Mating Inducing conditions. The Observed log odds ratio is obtained from cross-tabulation of the binding targets in the two conditions, and is equal to 4.56.

**Table 4:** Inferred positive specific agreement for target genes of Ste12 in the Rich Medium and Mating Inducing conditions.

	**FNR**					
	
		**0.20**	**0.25**	**0.30**	**0.35**	**0.40**
	
**FPR**	**0.001**	0.50	0.54	0.57	0.60	0.63
	
	**0.002**	0.55	0.58	0.60	0.62	0.64
	
	**0.003**	0.57	0.59	0.61	0.63	0.64
	
	**0.004**	0.59	0.60	0.62	0.64	0.65
	
	**0.005**	0.61	0.62	0.64	0.66	0.67

Inferred positive specific agreement values for different values of false positive rate and false negative rate for the binding targets of Ste12 in the Rich Medium and Mating Inducing conditions. The Observed positive specific agreement is obtained from cross-tabulation of the binding targets in the two conditions, and is equal to 0.38.

**Figure 8:**
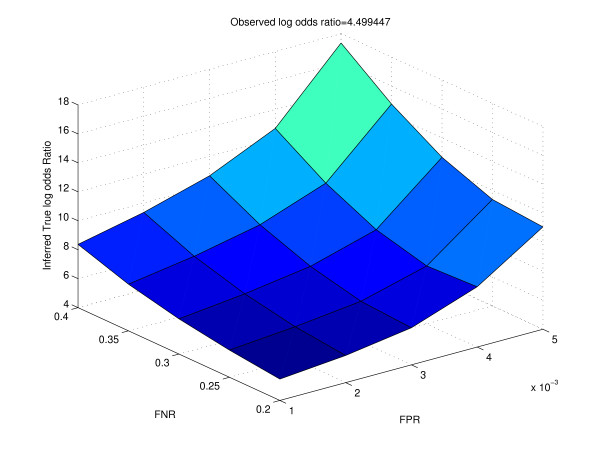
**Surface of the inferred true log odds ratio for different values of false positive rate and false negative rate for real data**. Observed log odds ratio is obtained from cross-tabulation of the binding targets of Ste12 under the pseudohyphal condition in *S. cerevisiae *and *S. mikatae *respectively.

**Figure 9:**
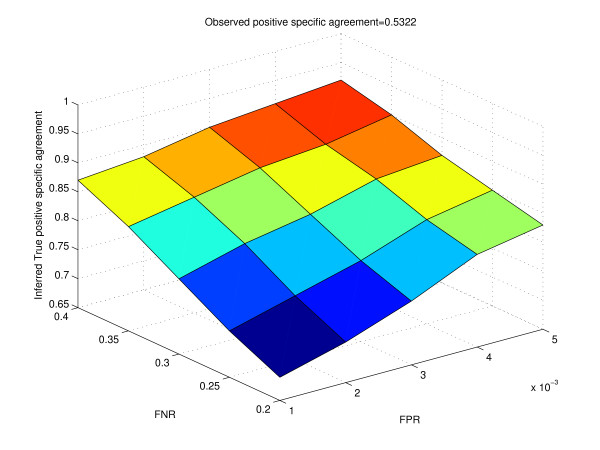
**Surface of the inferred positive specific agreement for different values of false positive rate and false negative rate for real data**. Observed positive specific agreement value is obtained from cross-tabulation of the binding targets of Ste12 under the pseudohyphal condition in *S. cerevisiae *and *S. mikatae *respectively.

We also analyzed the results of ChIP-chip experiments performed by Borneman *et al. *[8]. We obtained the counts of genes which were the binding targets of the regulators Ste12 and Tec1 in one, two and all three species. We repeated our analysis as described in the previous paragraph for a pair of species (Tables 5, 6 and 7; Figures 8 and 9). Here too, we notice a considerable difference between the observed and inferred outcomes as the false positive rate and false negative rate increases. For example, for a false positive rate of 0.001 and a false negative rate of 0.2, compared to the observed log odds ratio of 4.50, the inferred true log odds ratio is 5.48; however, for a false positive rate of 0.005 and a false negative rate of 0.4, the inferred true log odds ratio is as high as 17.36. Further, we attempted to test the notion that genes falling under similar functional categories tend to be the conserved binding targets across the three species. We listed all the orthologous genes in Yeast. For all these genes, we used SGD GO Slim finder  to categorize the genes into broad functional categories. For the top categories which contained the largest number of genes, we cross-tabulated the genes and created a 2 by 2 contingency table based on counts of the genes which are binding targets (Tables 8, 9, 10, 11). For the genes falling in major categories, we notice that the log odds ratios are considerably high, indicating considerable degree of binding conservation. For example, for the 565 genes found to be enriched for Hydrolase activity, 551 were not the binding targets of Ste12 in either *S. cerevisiae *or *S. mikatae*. Of the 14 genes which were the binding targets in at least one of the two species, 5 (YNL053W, YDR452W, YGL163C, YIL118W, YMR305C) were the binding targets in both species. Of the remaining 9 genes, 5 (YIR027C, YER133W, YNL180C, YOR049C, YDL047C) were targets in only *S. cerevisiae*, while 4 (YNL141W, YHR005C, YOR126C, YOL011W) were targets in only *S. mikatae*.

**Table 5:** Inferred true proportions of target genes of Ste12 under the pseudohyphal condition in S. cerevisiae and S. mikatae.

	**FNR**				
	**0.20**	**0.25**	**0.30**	**0.35**	**0.40**
	
**FPR = 0.001**	0.956	0.954	0.953	0.952	0.950
	0.006	0.005	0.003	0.002	0.001
	0.015	0.014	0.013	0.012	0.011
	0.023	0.026	0.030	0.034	0.038

	**0.20**	**0.25**	**0.30**	**0.35**	**0.40**
	
**FPR = 0.002**	0.958	0.957	0.956	0.954	0.953
	0.005	0.004	0.002	0.001	0
	0.014	0.013	0.012	0.011	0.010
	0.023	0.026	0.030	0.034	0.038

	**0.20**	**0.25**	**0.30**	**0.35**	**0.40**
	
**FPR = 0.003**	0.961	0.960	0.958	0.957	0.955
	0.003	0.002	0.001	0	0
	0.012	0.012	0.011	0.010	0.008
	0.024	0.026	0.030	0.033	0.037

	**0.20**	**0.25**	**0.30**	**0.35**	**0.40**
	
**FPR = 0.004**	0.964	0.962	0.961	0.959	0.957
	0	0	0	0	0
	0.011	0.010	0.009	0.008	0.007
	0.024	0.026	0.029	0.032	0.036

	**0.20**	**0.25**	**0.30**	**0.35**	**0.40**
	
**FPR = 0.005**	0.966	0.965	0.963	0.961	0.959
	0	0	0	0	0
	0.10	0.009	0.008	0.007	0.006
	0.024	0.026	0.029	0.032	0.035

For different values of false positive and false negative rates, the four inferred proportions of the target genes of Ste12 under the pseudohyphal condition in *S. cerevisiae *and *S. mikatae *are tabulated. The observed vector of proportions is (0.959, 0.010, 0.017, 0.015)^*t*^.

**Table 6:** Inferred true log odds ratios for target genes of Ste12 under the pseudohyphal condition in S. cerevisiae and S. mikatae.

	**FNR**					
	
		**0.20**	**0.25**	**0.30**	**0.35**	**0.40**
	
**FPR**	**0.001**	5.48	5.88	6.43	7.22	8.37
	
	**0.002**	5.88	6.26	6.93	7.92	9.35
	
	**0.003**	6.56	6.79	7.60	8.87	10.65
	
	**0.004**	8.16	8.00	8.90	10.51	12.69
	
	**0.005**	11.07	11.36	12.67	14.75	17.36

Inferred true log odds ratio values for different values of false positive rate and false negative rate for the binding targets of Ste12 under the pseudohyphal condition in *S. cerevisiae *and *S. mikatae*. The Observed log odds ratio is obtained from cross-tabulation of the binding targets in the two organisms, and is equal to 4.50.

**Table 7:** Inferred positive specific agreement values for target genes of Ste12 under the pseudohyphal condition in S. cerevisiae and S. mikatae.

	**FNR**					
	
		**0.20**	**0.25**	**0.30**	**0.35**	**0.40**
	
**FPR**	**0.001**	0.69	0.73	0.78	0.83	0.87
	
	**0.002**	0.72	0.76	0.81	0.85	0.88
	
	**0.003**	0.76	0.79	0.83	0.87	0.90
	
	**0.004**	0.81	0.83	0.86	0.88	0.91
	
	**0.005**	0.83	0.85	0.87	0.90	0.92

Inferred positive specific agreement values for different values of false positive rate and false negative rate for the binding targets of Ste12 under the pseudohyphal condition in *S. cerevisiae *and *S. mikatae*. The Observed positive specific agreement is obtained from cross-tabulation of the binding targets in the two organisms, and is equal to 0.53.

**Table 8:** Cross-tabulation of orthologous genes in functional category Hydrolase activity.

	*S. mikatae*		
	
		0	1
	
*S. cerevisiae*	0	551	4
	
	1	5	5

Cross-tabulation of orthologous genes in Yeast which fall into the GO Slim category **Hydrolase activity**, based on whether they are the binding targets of Ste12 in *S. cerevisiae *and *S. mikatae *respectively. We notice that there is a significant association, with *log odds ratio *= 4.93 and *p*_*pos *_= 0.53, indicating conservation of the binding targets across the two organisms.

**Table 9:** Cross-tabulation of orthologous genes in functional category Tranferase activity.

	*S. mikatae*		
	
		0	1
	
*S. cerevisiae*	0	491	7
	
	1	5	5

Cross-tabulation of orthologous genes in Yeast which fall into the GO Slim category **Transferase activity**, based on whether they are the binding targets of Ste12 in *S. cerevisiae *and *S. mikatae *respectively. We notice that there is a significant association, with *log odds ratio *= 4.25 and *p*_*pos *_= 0.45, indicating conservation of the binding targets across the two organisms.

**Table 10:** Cross-tabulation of orthologous genes in functional category Protein binding.

	*S. mikatae*		
	
		0	1
	
*S. cerevisiae*	0	338	3
	
	1	5	3

Cross-tabulation of orthologous genes in Yeast which fall into the GO Slim category **Protein binding**, based on whether they are the binding targets of Ste12 in *S. cerevisiae *and *S. mikatae *respectively. We notice that there is a significant association, with *log odds ratio *= 4.21 and *p*_*pos *_= 0.43, indicating conservation of the binding targets across the two organisms.

**Table 11:** Cross-tabulation of orthologous genes in functional category Transporter activity.

	*S. mikatae*		
	
		0	1
	
*S. cerevisiae*	0	225	3
	
	1	6	4

Cross-tabulation of orthologous genes in Yeast which fall into the GO Slim category **Transporter activity**, based on whether they are the binding targets of Ste12 in *S. cerevisiae *and *S. mikatae *respectively. We notice that there is a significant association, with *log odds ratio *= 3.91 and *p*_*pos *_= 0.47, indicating conservation of the binding targets across the two organisms.

## Conclusion

In this article, we have studied the effect of false positives and false negatives in the analysis and interpretation of ChIP-chip data. We have derived a relationship between the observed and the underlying true binary outcomes. Given the observed binary outcome of an experiment, we have developed an EM based approach to infer the underlying true binary outcome for given values of false positive and false negative rates.

A common limitation with finding binding targets from ChIP-chip data is that typically an arbitrary threshold, e.g. 0.001, is applied to the data, and all genes with p-values less than this threshold are considered binding targets. The false positive rate and false negative rate for the binding data change with the threshold applied [5]. Datta and Zhao [11] proposed a statistical procedure to determine the binding targets without imposing a simple threshold to the ChIP-chip data. However, their approach relies on accurate inference of false discovery rate [12], which is a non-trivial task.

To summarize data in contingency tables we utilized two commonly used metrics – *Odds Ratio *and *Positive specific agreement*. Both these metrics are widely used to study dependency among categorical variables. Since we are interested in quantifying association among two categorial variables, i.e. whether there is association across two different conditions/species, the *Odds Ratio *and *Positive specific agreement *are appropriate metrics of interest. In our simulation, we used the chi-squared test of independence to test the null hypothesis that the binding targets are independent. Instead of using the chi-squared test, we could also use the Fisher's exact test to test the independence assumption on the two-way contingency table. The resulting p-values from both tests indicate the statistical evidence against the independence assumption. However, they do not provide a meaningful summary of the degree of dependence as they are also dependent on the sample size.

In general, for independently performed real world experiments, such as two separate ChIP-chip experiments, the independence assumption of equation (9) should hold. This is because we can assume that data points in a particular experiment are independent identically distributed random variables. However, for experiments with closely associated results, it is possible that the false positive and false negative data points for the experiments are not entirely independent. This could result in an under-estimation of the underlying association after the EM procedure.

Due to the limited degrees of freedom of the data, our EM algorithm cannot be used to estimate the false positive rate and false negative rate in experimental data. At each step in the EM algorithm, we estimate three parameters, and we have three equations to solve for them. If we also wish to estimate the false positive and false negative rates, we would have two additional parameters, but the number of equations would still be three. This would lead to an identifiability problem.

We initialized the EM algorithm by different initial estimates of the parameters. For each initial estimate of the parameters, the algorithm converged. The convergence criteria for the EM algorithm require that the log likelihood of the parameters *l*(**b**|**g**) be continuous and differentiable in the parameter space. Unfortunately, the M-step of our algorithm does not have a closed form. Hence, it is difficult to evaluate the gradient of the log likelihood function.

Harbison *et al. *performed their ChIP-chip experiments using microarrays consisting of spotted polymerase chain reaction (PCR) products representing all the intergenic regions of *Saccharomyces cerevisiae*. To obtain the binding targets a p-value threshold was applied to the binding intensities associated with the probes. One of the drawbacks of PCR based arrays is the low resolution of the DNA elements in the microarray chip. For PCR arrays designed for Yeast, the typical resolution achieved is less than 1 kb. In recent years, high density oligonucleotide arrays, comprising of large numbers (40, 000 to more than 6, 000, 000) of short oligonucleotides have been utilized for ChIP-chip studies [13-16]. A number of statistical algorithms have also been developed to determine the binding targets from such large scale tiling arrays [17-20]. Borneman *et al. *used high density oligonucleotide arrays to perform their experiment. The binding targets were obtained using Tilescope [21]. Since they report the target genes in each organism, we simply used their results to obtain the counts of target genes in each of the three organisms.

Our analysis can be applied to any experimental setting with binary outcomes. However, for the sake of simplicity, we have illustrated its application for ChIP-chip experiments. By applying our algorithm to ChIP-chip data from Harbison *et al. *and Borneman *et al.*, we observe that for different values of the false positive and false negative rate, the observed and true metrics for the binary data can differ quite dramatically. However, we notice that when the true log odds ratio is greater than 4, i.e. there is a significant degree of association among the binding targets across conditions/species, such differences in the observed and true metrics would not change our inference. On the other hand, our simulation results indicate that when the true odds ratio is close to 1, i.e. for cases when the underlying association is marginal, moderate values of false positive and false negative rates (0.01 and 0.2 respectively) may not be able to provide conclusive evidence of any underlying association or independence.

## Authors' contributions

DD performed data analysis and drafted the manuscript. HZ conceived and guided the study. Both authors read and approved the final manuscript.
